# Analysis of gene expression in early seed germination of rice: landscape and genetic regulation

**DOI:** 10.1186/s12870-022-03458-3

**Published:** 2022-02-17

**Authors:** Haoxuan Li, Xiaozheng Li, Guanjie Wang, Jianhua Zhang, Guanqun Wang

**Affiliations:** 1 Guangdong Key Laboratory of Plant Epigenetics, College of Life Sciences and Oceanography, Shenzhen University, Shenzhen, 518060 China; 2 School of Life Sciences and State Key Laboratory of Agrobiotechnology, The Chinese University of Hong Kong, Shatin, Hong Kong, China; 3 Department of Biology, Hong Kong Baptist University, Kowloon, Hong Kong, China; 4 College of Life Sciences, Jilin Agricultural University, Jilin, China

**Keywords:** Oryza sativa, Early seed germination, Physiological characteristic, Transcriptome, Transcription factors

## Abstract

**Background:**

Seed germination is a crucial process, which determines the initiation of seed plant life cycle. The early events during this important life cycle transition that called early seed germination is defined as initially water uptake plus radicle growing out of the covering seed layers. However, a specific genome-wide analysis of early seed germination in rice is still obscure.

**Results:**

In this study, the physiological characteristics of rice seed during seed germination are determined to define key points of early seed germination. Transcriptome analyses of early phase of seed germination provided deeper insight into the genetic regulation landscape. Many genes involved in starch-to-sucrose transition were differentially expressed, especially alpha-amylase 1b and beta-amylase 2, which were predominantly expressed. Differential exon usage (DEU) genes were identified, which were significantly enriched in the pathway of starch and sucrose metabolism, indicating that DEU events were critical for starch-to-sucrose transition at early seed germination. Transcription factors (TFs) were also dramatic expressed, including the abscisic acid (ABA) responsive gene, *OsABI5,* and gibberellic acid (GA) responsive genes, *GAI*. Moreover, *GAI* transactivated GA responsive gene, GAMYB in vivo, indicating a potential pathway involved in early seed germination process. In addition, CBL-interacting protein kinase (CIPK) genes, such as CIPK13, CIPK14 and CIPK17 were potentially interacted with other proteins, indicating its pivotal role at early seed germination.

**Conclusion:**

Taken together, gene regulation of early seed germination in rice was complex and protein-to-gene or protein-to-protein interactions were indispensable.

**Supplementary Information:**

The online version contains supplementary material available at 10.1186/s12870-022-03458-3.

## Background

Direct seeding of rice is a popular cultivation manner in rural areas. However, this manner is hindered, because of high requirements on seed vigor under different stress conditions in wild. Seed germination process mainly contain three phases: absorbing water quickly and enhanced metabolic; absorbing water slowing, mRNA synthesis, the modulating of hormones, subsequently followed with cell division, radicle elongation and germination; accelerated water absorption, growth rate of radicle and germ until the formation of complete seedlings. Hence, seed germination is the result of embryo activation through a mechanism containing morphological and physiological alterations. The preparatory work, such as water uptake, which resulted in the expansion and elongation of seed embryo is needed before germination. As a matter of fact, emergence of the embryonic axis, usually referring to radicle growing out of the covering seed layers, is defined as the completion of seed germination [1–3].

Notably, seed germination in rice has been demonstrated to be regulated by various genes [4–8]. Seed dormancy is universal in most angiosperms at maturity, which should be break down before germination (Bewley, 1997). Dormancy is a complex developmental process and many factors are involved in regulating seed dormancy, including some plant hormones, such as abscisic acid (ABA), auxin, gibberellic acid (GA), and ethylene [1–3, 9–14]. However, the mechanisms of dormancy holding and breaking remain obscure, which is critical in the crop production [15]. The transition from dormancy to germination in seeds is a key physiological process during the lifecycle of plants. Phases from initially water uptake to radicle growing out of the covering seed layers was the most important stage in seed germination, thereafter, acquiring a comprehensive knowledge of this stage is necessary. Previous studies of proteomics on rice seeds germination were mainly focused on seeds germination time-course from the dry seeds to the formation of complete seedlings [16–19], whereas gene expression profile of seeds germinated in the most important stage compared with the water uptake stage is still obscure. In this study, we tracked the physiological characteristics of rice seeds germination from the dry seeds to seedlings, and found the key time point of seed germination initiation. Time point of seeds starting to germinate is essential for understanding the multi-regulations during seeds germination.

## Results

### Physiological characteristics of seed germination at the early stages

**Fig. 1:**
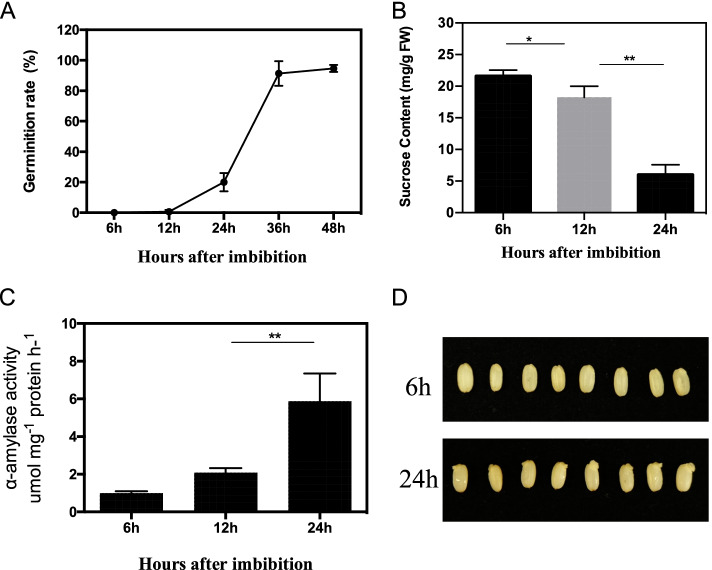
Physiological characteristic of seed germination. **A** Germination rate of rice seeds after imbibition. **B** Sucrose content of imbibed seeds at 6 h (6 h) after imbibition, 12 h after imbibition and 24 h after imbibition. **C** alpha-amylase activity of imbibed seeds at 6 h after imbibition, 12 h after imbibition and 24 h after imbibition. **D** Seeds imbibed after 6 h and 24 h. Values are means (± SD) of three replicates. Significant differences were determined using student t test: *P < 0.05. **P < 0.01

To gain insight into the molecular mechanism of rice seed (*Oryza* sativa. japonica) germination initiation, we performed the seed germination experiment to find out the time of starting germination. We observed that seeds begin to germinate at 24 h after imbibition, and then the germination rate increased saltatory at 36 h (Fig. 1A), suggesting that the time point of 24 h is essential for studying the mechanism of germination initiation. Starch degraded into sucrose by amylase enzyme is important to support seeds germination. Then, we measured the sucrose content at 6 h, 12 h and 24 h, and the results showed dramatic decrease of sucrose content at 24 h, compared with that of 6 h and 12 h, while the sucrose content of 12 h was only slightly decreased compared with that of 6 h (Fig. 1B). Moreover, the alpha-amylase activities were increased from the 6 h to 24 h, and the activities of 24 h was more than four times of that 6 h (Fig. 1C), which further indicated time point of 24 h and 6 h are the good sample time for investigation of the germination activation process. After that, we collected the germinating seeds at 6 h and 24 h after imbibed in the pure water. RNA sequencing (RNA-seq) libraries generated from total RNA with three independent replicates at 6 h and 24 h time points were deployed for the following analysis [20].

### Transcriptome profile of seed germination at early stages

**Fig. 2:**
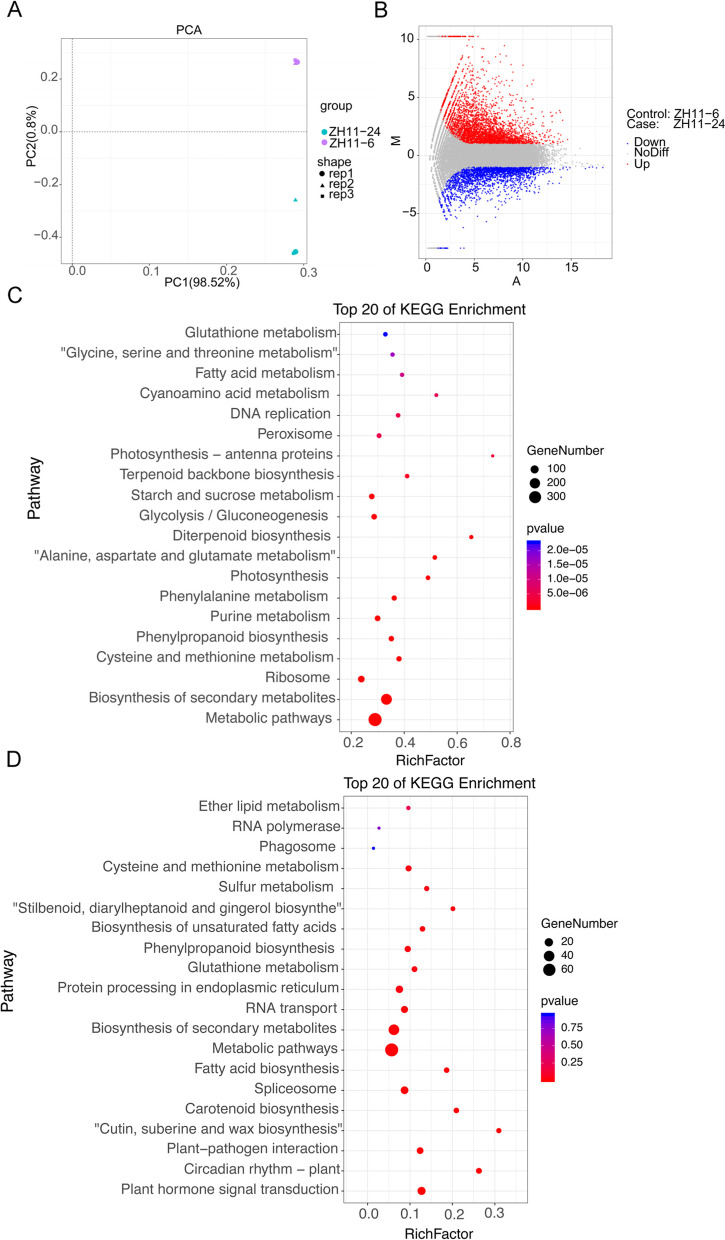
Transcriptome profiling of early seed germination at 6 h (6 h) after imbibition and 24 h (24 h) after imbibition. **A** Principal component analysis of imbibed seeds at 6 h and 24 h. **B** MA plot of the imbibed seeds at 6 h and 24 h showing the up, down and none differential (NoDiff) genes. **C** KEGG pathway enrichment on the up regulated genes at 24 h compared with that of 6 h. **D** KEGG pathway enrichment on down regulated genes at 24 h compared with that of 6 h

After mapping, more than 94.37% clean reads were mapped to gene regions, in which larger than 97.23% clean reads were mapped to the exon regions. In addition, the percentage of uniquely mapped clean reads was larger than 91.86 among all the libraries. Principal component analysis (PCA) based on all the genes detected among the six libraries were plotted to illustrate the similarity of the transcriptomes at 6 h and 24 h (Fig. 2A). The results revealed a long distance between the samples at 6 h and 24 h. Then, we analyzed the differentially expressed genes (DEGs), and a total of 8701 DEGs were identified, among which 5768 DEGs were up-regulated and 2933 were down-regulated in germinating seeds at 24 h compared to 6 h (Fig. 2B and Supplementary Table S1). To figure out the role of DEGs in seed germination initiation process, we performed the KEGG pathway enrichment on the up-regulated and down-regulated genes, respectively (Fig. 2C and D). In terms of the up-regulated DEGs, the top three enriched KEGG pathway categories were “Metabolic pathways”, “Biosynthesis of secondary metabolites” and “Ribosome”. There were also 33 up-regulated DEGs gathered in “starch and sucrose metabolism”, and 27 up-regulated DEGs enriched in “Plant hormone signal transduction” (Fig. 2C). In contrast, the down-regulated DEGs were significantly enriched in the pathway of “Plant hormone signal transduction” and “Spliceosome”. (Fig. 2D). Furthermore, a total of 51 up-regulated genes and 17 down-regulated genes were enriched in the pathway of “starch and sucrose metabolism”, which involved in sucrose synthesis and starch degradation, suggesting the potential role of those DEGs in modulating seed germination at the germination activation stage. (Fig. S1).

### DEGs involved in the starch degradation and sucrose synthesis

**Fig. 3:**
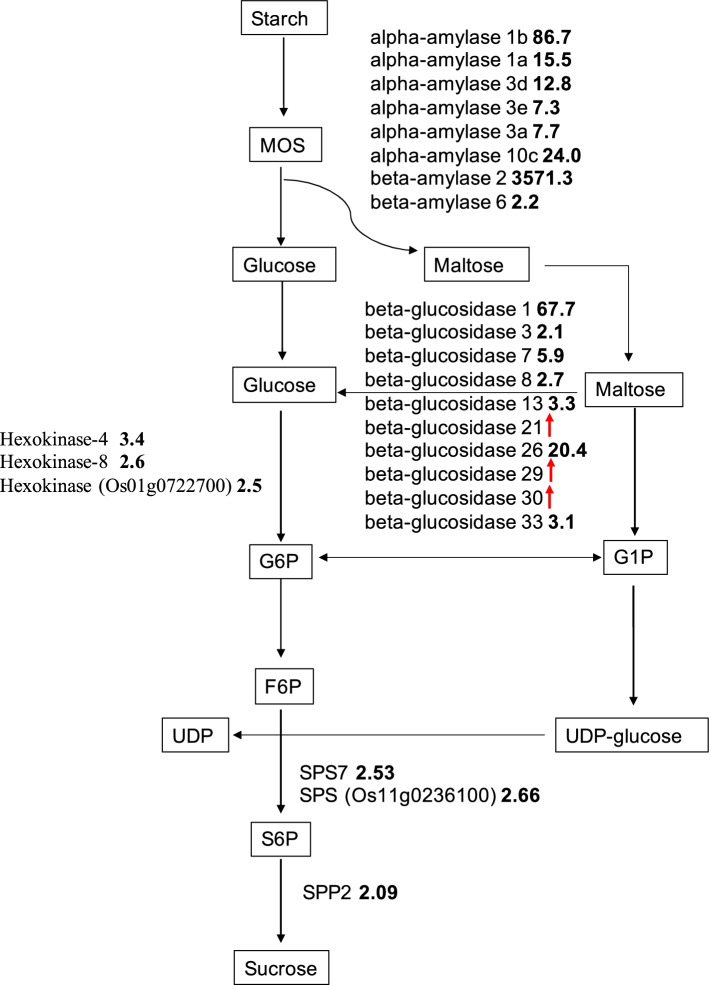
Expression patterns of the genes involved in the conversion of starch to sucrose at 24 h (24 h) after imbibition compared with that of 6 h (6 h) after imbibition. The ratio indicates the fold difference in the respective gene transcript levels of 24 h compared to 6 h. The red arrow represents genes that only expressed at 24 h

Developing embryo during seed germination depends entirely upon carbohydrate reserves remobilized from starch granules stored predominantly in the endosperm [21]. Therefore, figuring out which genes responded at early seed germination stages is essential. Up-regulated DEGs involved in the conversion from starch into sucrose were plotted in the Fig. 3. A total of 6 alpha-amylase genes were significantly increased at 24 h with large fold changes, and 2 beta-amylase also showed enhanced expression levels (Fig. 3). Notably, beta-amylase 2 displayed predominant expression levels at 24 h, which was 3571.3-times of that at 6 h, suggesting its dominant role in starch break down process at the germination initiation phase. A total of ten beta-glucosidase genes, responsible for the conversion from maltose to glucose, were significantly up regulated at 24 h, among which three genes were only expressed at 24 h (Fig. 3). In addition, genes encoding hexokinase were also up regulated, which facilitated glucose converted into glucose-6-phosphate (G6P). After that, the sucrose was then resynthesized, which catalyzed by the SPS and SPP. In this study, two genes encoding SPS and one gene encoding SPP were enhanced at 24 h. However, some genes involved in the starch-sucrose conversion were down regulated at 24 h. For example, beta-amylase PCT-BMYI (Os03g0141200) was 0.28 times to that of 6 h, indicating its role in starch degradation at pre-germination phase. Five genes encoding beta-glucosidase (Os01g0940700, Os04g0513100, Os05g0366600, Os08g0525800, Os10g0473400) showed higher expression levels at 6 h, which modulated starch degradation at pre-germination phase. Additionally, gene encoding hexokinase-1 was also down regulated at 24 h.

### Hormone signal transduction in germinating seed

**Fig. 4:**
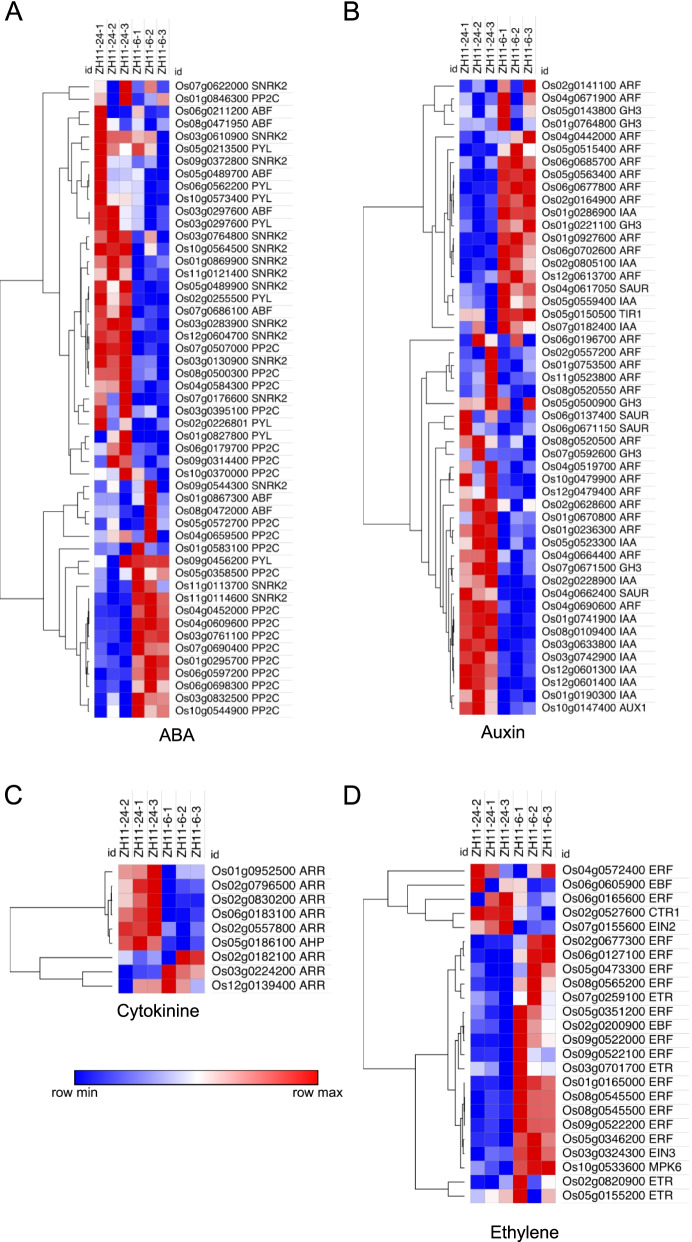
Expression levels of genes involved in the hormone signal transduction pathway. **A** Heatmap of genes involved in the ABA signal transduction pathway. **B** Heatmap of genes involved in the auxin signal transduction pathway. **C** Heatmap of genes involved in the cytokinine signal transduction pathway. **D** Heatmap of genes involved in the ethylene signal transduction pathway. Blue color represents low expression level, and red color represents high expression level

Seed germination contributes greatly to plant production, which is a complex process involving various signal transduction pathways [22]. First, the phytohormones ABA and GA antagonistically regulate seed germination, induce seed dormancy and break seed dormancy. The genes involved in ABA signal transduction and the GA signaling pathway also influence seed dormancy. Then, we analyzed the expression pattern of the relative genes belonging to the ABA signal transduction, and the KEGG analysis showed that a large majority of genes encoding the ABA receptors of pyrabactin resistance 1/PYR1-like (PYL/PYR), ABRE binding factor (ABF) and SNF1-related protein kinases (SNRK) were significantly up regulated at 24 h, whereas protein phosphatases type 2C (PP2C) genes were down regulated at this time point (Fig. 4A). In contrast, genes encoding transcription factors involved in the GA signal transduction pathway were down regulated at 24 h (Fig. S2A). Genes involved in the ethylene signal transduction pathway were also decreased at 24 h compared with that at 6 h (Fig. 4D). It’s demonstrated a crosstalk between auxin and ABA involved in rice seed germination process (He et al., 2020). In our study, genes responsible for auxin signal transduction pathway was complex that expression levels of genes encoding the AUX1 was enhanced at 24 h, while genes encoding GH3 were only up regulated at 6 h (Fig. 4B). In addition, genes responsible for IAA and SAUR were either up regulated or down regulated at 24 h compared to 6 h (Fig. 4B). Genes involved in the cytokinine, salicylic acid (SA) and jasmonic acid (JA) signal transduction were also up or down regulated at 24 h (Fig. 4C and Fig. S2B, C). ABI5 gene was demonstrated to negatively regulate seed germination in plants (Wang et al., 2011; Albertos et al., 2015; He et al., 2020). In this study, the expression levels of ABI5 was decreased at 24 h, suggesting its role in promoting seed germination (Fig. S2D).

### Changes in the expression profile of TFs during seed germination initiation

**Fig. 5:**
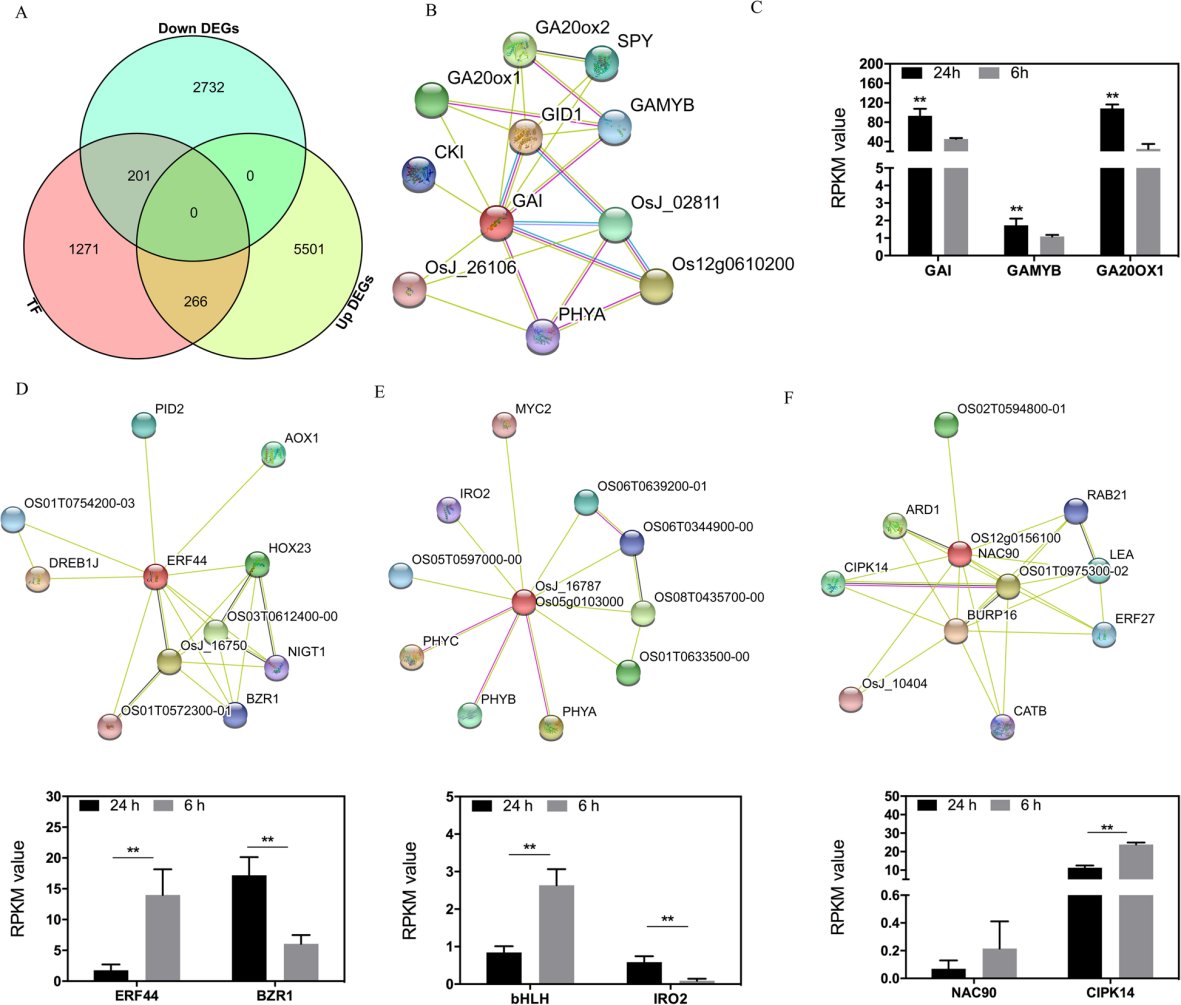
Differentially expressed transcription factors (TFs) at 24 h (24 h) after imbibition compared with that of 6 h (6 h) after imbibition. **A** Venn diagram showed the number of differentially expressed TFs. **B** TF of GAI was predicted to interact with GA synthesis genes, including GA20ox1 and GA20ox2, and interact with GA responsive gene, GAMYB. **C** The expression levels of GAI, GA20OX1 and GAMYB, and transient expression assay of GAI transcription factor in vivo*.* Values are means (± SD) of three biological replicates. **D** TF of ERF44 was potentially interacted with BZR1. E, TF of bHLH (Os05g0103000) was potentially interacted with IRO2. F, TF of NAC90 was potentially interacted with CIPK14. Transcript levels are means (± SD) of three replicates. Significant differences were determined using student t test: *P < 0.05. **P < 0.01

The time point of seed starting to germination led to a large number of differentially expressed TFs which belong to multiple TFs families. A total of 266 up-regulated TFs (involved 43 TFs families) and 201 down-regulated TFs (contained 35 TFs families) were detected at 24 h compared with that of 6 h (Fig. 5A). The up-regulated TFs were significantly enriched in the GO terms of nitrogen compound metabolic process, nucleic acid metabolic process, and biosynthetic process. We also performed the KEGG analysis on up-regulated TFs, in which 9 TFs were significantly enriched in the plant hormone signal transduction. Notably, one TFs, GAI (Os03g0707600) was predicted to interact with genes involved in the GA signal transduction pathway, including the GA biosynthesis genes GA20ox1 and GA20ox2, and GA responsive genes GAMYB (Fig. 5B and Fig. S3). Furthermore, the expression level of *GAMYB* and *GA20ox1* were also up regulated at this time point, indicating the potential role of elevated GAI transactivating the expression of *GAMYB* and *GA20OX1* (Fig. 5C). We performed the luciferase assay using transient expression system in rice protoplasts and found that GAI transactivated the expression of *GAMYB* expression in vivo, indicating the potential role of GAI in controlling seed germination in rice (Fig. 5C). Another TF belonging to MYB family (Os01g0524500) responses to GA and sucrose, indicating its potential roles in the utilization of sucrose to control the seed germination process.

For the down-regulated TFs, a total of 201 down-regulated TFs were classified into thirty different TF families, including the families of WRKY, bHLH, ERF, NAC, and MYB. However, there was no significant enriched KEGG pathways amongst the down-regulated TFs. To be noted that one of the above down-regulated TFs, ethylene-responsive transcription factor ABI4 responses to the sucrose status, which was involved in the abscisic acid-activated signaling pathway to negatively regulate the seed germination. To investigate the potential gene regulatory network controlled by TFs, we examined the interacting proteins of the above down-regulated TFs. Many of the down-regulated TFs were potentially co-expressed with other proteins. The Os08g0565200 gene encoding a ERF44 TF was dramatic down regulated at 24 h (Fig. 5D). ERF44 was predicted to co-express with another TF, BZR1, which was the feedback inhibition of BR biosynthesis (Fig. 5D). Besides, the expression levels of BZR1 were significantly increased at 24 h, indicating that ERF44 might possess the inhibition effect on the transcription of BZR1. A bHLH gene (Os05g0103000), displayed decreased expression level at 24 h, was also revealed to interact with other proteins, such as the protein of IRON-RELATED TRANSCRIPTION FACTOR 2 (IRO2) (Fig. 5E and Fig. S3). Furthermore, some down-regulated TFs might also possess transactivation effect on its co-expressed proteins. For instance, down-regulated NAC90 encoded by Os12g0156100, was revealed to interact with CBL-interacting protein kinase 14 (CIPK14), which showed reduced expression levels at 24 h (Fig. 5F and Fig. S3).

### Differential exon usage (DEU) analysis in seed germination process

**Fig. 6:**
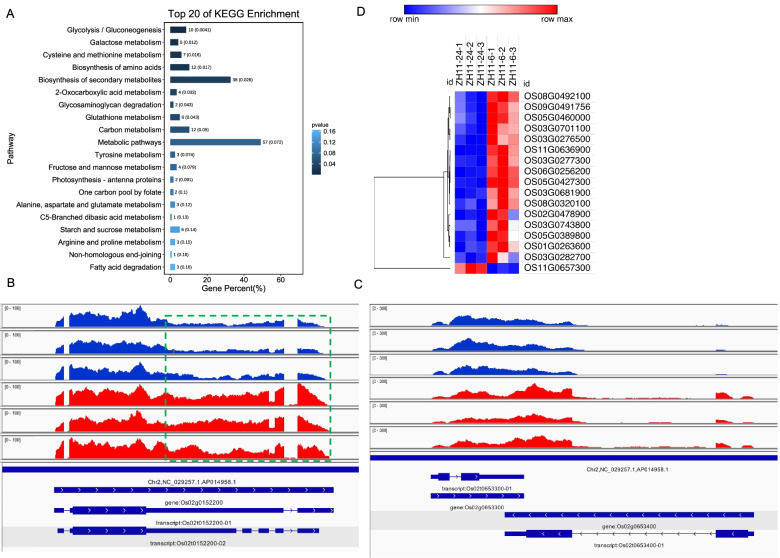
Differential exon usage (DEU) at 24 h after imbibition compared with that of 6 h after imbibition. **A** KEGG analysis of DEU genes between 24 h after imbibition and 6 h after imbibition. **B** Example of increased DEU gene (OS01g0765000) at 24 24 h after imbibition. **C** Example of increased DEU gene (OS02g0653400) at 24 24 h after imbibition. Blue color shows the expression level of 6 h, while red color shows the expression level of 24 h. **D** Most of splicing factors at 24 h were down regulated. Blue color represents low expression level, and red color represents high expression level

Differential exon usage (DEU) can lead to different functional gene products arising from a single genomic locus, which adds greatly to the diversity of gene products encoded by the genome [23]. Differential exon usage was studied to reveal alternative splicing and transcription events, because that exon usage is synchronous to exon spliced into the transcriptome, which was more directly than the alternative splicing. In this study, a total of 80 DEU events occurred in the protein coding genes were significantly classified between the 24 h and 6 h (Supplementary Tables S2 and S3). And the KEGG analysis revealed that most of the DEU genes were significantly enriched in the pathways of metabolic and biosynthesis of secondary metabolites, while 6 DEU genes were enriched in the starch and sucrose metabolism pathway to support seed germination (Fig. 6A). For example, two elevated DEU genes, OS01g0765000 and OS02g0653400, encoding Cytidine/deoxycytidylate deaminase family protein and transferase family protein, respectively, showed a similar expression pattern to it’s orthologous genes of Arabidopsis during seed germination (Fig. 6B, C and Fig. S4A, B) [24]. In addition, miRNA-targets interaction usually repressed target gene expression. To explore the possibility that miRNAs target to the differentially expressed genes which leading to the DEUs, we performed the prediction targets of differential expressed DEUs (Supplementary Table S4). A total of 85 miRNAs were predicted to target the 16 DEU genes, among which some of DEU genes were targeted by different miRNAs. Previously study also predicted the targets of identified miRNAs during early seed germination [25]. According to the reported miRNA-targets prediction, we found 9 DEU genes were targeted by 9 expressed miRNAs (Supplementary Table S4). Those miRNAs might be responsible for the DEU events in the early seed germination phase.

Gene expression changes in splicing factors also play key roles in guiding plant developmental processes [26]. Differential splicing factor expression can result in alternative splicing differences [27]. Therefore, the expression changes of splicing factors played roles in mediating the alternative splicing events. In this study, a total of 17 splicing factors were either up or down regulated during early seed germination process. Interestingly, there were only 1 increased splicing factors in 24 h, while 16 elevated splicing factors at 6 h (Fig. 6D and Fig. S4C).

### Protein interaction prediction based on the DEGs

**Fig. 7:**
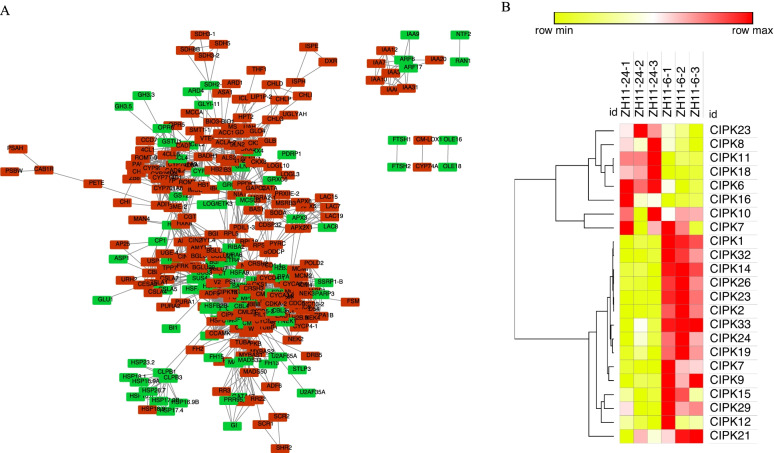
Protein–protein interaction prediction based on the differentially expressed genes between 24 h after imbibition and 6 h after imbibition. **A** Protein–protein network. Red blocks are genes increased at 24 h, and green blocks are genes increased at 6 h. **B** Heatmap of all the differentially expressed CIPK genes. Yellow color is the low expressed genes, while red is the high expressed genes

In total, 8701 DEGs were identified in the comparisons of 24 h_vs_6 h of the seeds after imbibition (Fig. 1), suggesting a complex gene regulatory network, for example, the protein–protein interactions. Therefore, protein–protein interaction network based on the DEGs were conducted to display the potential interactions which might be essential for the rice seed germination initiation. We identified 1648 pairs of protein–protein interactions with different interaction scores (Fig. 7A and Supplementary Table S5). It was demonstrated that ( CBL-interacting protein kinase) CIPKs were involved in the seed germination process, such as CIPK3 [28], CIPK31 [29] and CIPK15 [30, 31]. In this study, we found that 23 CIPK genes were differentially expressed, in which CIPK13, CIPK14 and CIPK17 were predicted to interacted with various proteins (Fig. 7B and Supplementary Table S6). Those candidate interaction pairs were mainly the protein families of CMLs, HSFs, and ETRs, indicating its potential role in initiating seed germination (Supplementary Table S6).

## Discussion

Seed germination is a complex process and is controlled by interactions of many internal factors [32]. The landscape of seed germination from initially water uptake to radicle growing out of the covering seed layers was essential for revealing comprehensive knowledge of this stage. First of all, figuring out the accurate transition time of seed germination from water uptake to radicle growing out was of interest. Previous evidence showed that the imbibed seeds followed three phases of water uptake: including phase I covered 6 h from the onset of imbibition, followed by a plateau phase (Phase II) to 24 h, and then the start of germination (Phase III) [1, 33]. Additionally, some other studies also investigated the initial imbibition of seed germination in rice at 8 h after imbibition compared with the dry seeds [34] and the embryo expression profiles during imbibition at dry seeds, 12 h and 24 h [35]. However, the sampling imbibition time of these studies only based on the water uptake status. In this study, we examined physiological characteristics of rice seeds germination, including the germination rate, sucrose content and amylase activities, from the dry seeds to seedlings, which were combined to determine the key phases of germination at early stages (Fig. 1). We found that the seeds started to germinate from 24 h after imbibition, and the sucrose content was significantly reduced at 24 h compared with that of 6 h, while it was negative consistent with the amylase activities (Fig. 1A, B, C). Taken together, the time point of 24 h is critical because it links Phase II and Phase III and determines a successful germination. Furthermore, the point of 6 h links the Phase I and Phase II that sustains a plateau condition. Thereafter, time point of water uptake at 6 h and germination starting time point at 24 h were the critical points for early seed germination in rice, which were valuable for investigating genetic landscape of early germination.

The starch in the endosperm serves as a nutrient source for seed germination and seedling establishment, which is catabolized upon the initiation of germination by hydrolytic enzymes secreted from the aleurone layer [36]. Thus, the starch to sucrose conversion is critical for the early seed germination. Literally, starch can be degraded via hydrolysis and phosphorolysis, which are catalyzed by several enzymes, including alpha‐amylase, beta‐amylase, alpha‐glucosidase, beta-glucosidase and starch phosphorylase (Preiss, 1982; Gallagher et al., 1997). Then, sucrose is synthesized by the enzymes of sucrose phosphate synthase (SPS), sucrose phosphate phosphatase (SPP) and sucrose synthase (SUS) (Whittingham et al., 1979; Wardlaw and Willenbrink, 1994; Isopp et al., 2000; Wang et al., 2017). In our study, we found that many genes encoding alpha-amylase, beta-amylase, beta-glucosidase, hexokinase, SPS, and SPP were highly expressed at 24 h. Especially, alpha-amylase 1b and beta-amylase 2 showed the highest expression levels, suggesting its primary roles in starch degradation during early seed germination phase (Fig. 3). In addition, beta-glucosidase 1 and beta-glucosidase 26 also displayed dramatic expression levels at 24 h compared with that of 6 h (Fig. 3). Genes responsible for sucrose synthesis, including SPS and SPP, were also increased at early seed germination (Fig. 3). Therefore, the above genes were critical for providing the energy source required by the seed germination initiation. However, some genes involved in the starch-sucrose conversion were highly expressed at 6 h, such as beta-amylase PCT-BMYI, beta-glucosidase and hexokinase-1, indicating its role in initiating the starch degradation at pre-germination phase.

Evidence showed that abscisic acid (ABA) and gibberellin (GA) have antagonistic roles in the regulation of seed germination [22, 37]. Endogenous ABA content reached a sufficient decrease at the end of phase I (6 h) during imbibition and continued to decrease until the end of phase II, which is a major prerequisite for the completion of germination [38–40]. ABA signal transduction pathway, responding to the ABA content, consists of four core components, and these proteins form a double-negative regulatory system, in which ABA binds to receptors of the PYR/PYL family and forms ternary complexes with clade A protein phosphatases type 2C (PP2Cs), thereby abrogating their inhibitory effects on SNF1-related protein kinases 2 (SnRK2.2/3/6), and leading to activation of the ABA signaling pathway. During the early seed germination, most of the core components were up regulated, whereas genes encoding PP2C proteins showed the opposite expression patterns compared with other components (Fig. 4A). GAs are also important during the early seed germination and counteract the ABA inhibition [41]. Transcripts of *GA20ox1*, critical for GA biosynthesis, accumulated during early germination (Fig. 5C), which was consistent with previous evidences [41]. It was demonstrated that Auxin and ABA had a crosstalk during rice seed germination process (He et al., 2020). Expression levels of genes involved in the auxin signal transduction pathway were either increased or reduced during early seed germination, indicating its complex regulatory network (Fig. 4B). Ethylene was demonstrated to counteract the ABA inhibition by interfering with ABA signaling, during the late phase of germination [42]. In contrast, amongst genes involved in the ethylene signal transduction, most of those genes were down regulated at 24 h, which suggested that ethylene might be also critical for early seed germination (Fig. 4D). In addition, genes responsible for the cytokine, SA and JA were differentially expressed during early seed germination, which might be pivotal for the preparation of seed germination (Fig. 4C and Fig. S2B, C).

It was demonstrated that CBL-interacting protein kinase (CIPK) was involved in the seed germination process, such as CIPK3 which interacted with calcium sensor CBL9 was a negative regulator of ABA response in germination in Arabidopsis [28]; CIPK31 was involved in germination in rice plants [29]; and CIPK15, a hallmark gene for anaerobic germination [30, 31]. Overall, 23 CIPKs genes were differentially expressed during early seed germination phase, among which three CIPKs genes, CIPK13, CIPK14 and CIPK17 might be pivotal for the preparation of the seed germination because of its complex interaction protein network (Fig. 7 and Supplementary Table S1). Therefore, the validation of the protein–protein interaction network of CIPK13, CIPK14 and CIPK17 is necessary for revealing the landscape of early seed germination in future studies.

In this study, a large number of DEGs identified during early seed germination might be regulated by the differentially expressed TFs (Fig. 2). It’s noteworthy that TFs, especially those responsive to the ABA signaling are pivotal for the seed germination. The ABI5 transcription factor, an important positive regulator of ABA signaling, was significantly reduced from 6 to 24 h (Fig. S2D), which was caused by reduced endogenous ABA [43]. In addition, some other TFs might play roles in early seed germination. Notably, up-regulated transcription factor of GAI (Os03g0707600) involved in the plant hormone signal transduction pathway potentially interacted with the GA responsive genes GAMYB (Fig. 5B), which involved in the GA signal transduction pathway. Furthermore, *GAMYB* was reported to modulate α-amylase gene expression in aleurone and flower development, which might also pivotal in early seed germination process [44]. In our work, we verified that the up-regulated TF of GAI transactivated the expression of *GAMYB* (Fig. 5C), which seems contradictory to the theory that GAI is a repressor of gibberellin (GA) signaling in rice [45, 46]. Therefore, genetic analysis of *GAMYB* regulated by GAI needs further investigation.

## Conclusions

We definite two accurate time points of early seed germination and subsequently, draw the comprehensive gene expression profiling, which revealing a more specific gene regulation landscape at early seed germination. In addition, an up-regulated TF, GAI might transactivate the GA responsive gene GAMYB, which provides a potential pathway of GAI mediated seed germination.

## Methods

### Plant growth conditions and seeds germination assay

Rice seeds Oryza sativa L. cv. Zhonghua 11 (ZH11), a wild type rice obtained from Professor Nenghui Ye belonging to Hunan Agricultural University, were used as the experiment materials. Guanqun Wang undertook the formal identification of the ZH11 seeds. Seeds were germinated in sterile petri dishes containing moistened paper towels under 28 °C in a dark growth chamber [38, 47]. Seeds with radical length larger than 1 mm were considered as the germinated seeds, and the germination rate was calculated in every 12 h until fully germinated with three biological replications. A total of forty seeds were included in each of the replicate. Seeds were rapidly sampled and frozen in liquid nitrogen and stored under -80 °C for further experiment.

### Sucrose content measurement

Sucrose content of seeds sampled at 6 h, 12 h and 24 h after imbibition were measured. Briefly, 0.5 g sampled seeds were ground into fine power on ice and transferred within 10 ml 80% (v/v) ethanol in a 15 ml centrifuge tube, which was followed with extraction in a water bath at 80 °C for 30 min. After then, the samples were centrifuged at 8000 g for 10 min to collect supernatant. The extraction process was repeated with three times. After the extraction, all the collected supernatant was diluted to a volume of 50 ml with distilled water. The sucrose content was measured by anthrone reaction as previously described [45].

### Amylase activity assay

Imbibed seeds at 6 h, 12 h and 24 h after imbibition were harvested for enzyme extraction as previously described [20]. The assay kit from Jiancheng, biotechcompany (Nanjing, China) was used in this study.

### RNA extraction and library construction

Total RNA of the germinating seeds sampled at 6 h and 24 h after imbibition were extracted with three replicates using RNeasy Plant Mini Kit (Qiagen, Valencia, CA). Non-strand specific libraries were constructed as previously described [45]. Then the prepared library was sequenced on the Illumina Hiseq4000 PE101 platform. After sequencing, the raw data was obtained by base calling and stored in fastq format. Reads Per Kilo bases per Million reads (RPKM) value were calculated by StringTie v1.3.3, which subsequently followed with differential expression analysis in Ballgown package [48, 49]. Genes with fold change (absolute value) > 2 and *P*-value < 0.05 were filtered as differentially expressed genes (DEGs).

### GO and KEGG pathway analysis

Functional classification of the DEGs was performed using Gene Ontology (GO) slims (http://bioinfo.cau.edu.cn/agriGO/). Kyoto Encyclopaedia of Genes and Genomes (KEGG) pathways (https://www.omicshare.com/tools/Home/Soft/seniorbubble) was deployed for enriched KEGG pathways analysis.

### Differential exon usage (DEU) analysis

DEXSeq is used to analysis exon usage from the RNA-seq data as previously reported [50]. Regarding to each exon, we calculate the reads number of each exon (Reads 1) and reads number of this exon mapped to the other exons in the same gene (Reads 2). Ratio of Reads 1/ Reads 2 is the value of exon usage. The different exon usage were regarded as the DEU.

### Protein–protein interaction prediction

Protein–protein interaction prediction was conducted based on the STRING10.0 (http://string-db.org) database. Protein–protein interaction was obtained based on DEGs. Then the Protein–protein interaction network was plotted in Cytoscape (www.cytoscape.org).

### Transient expression analysis of transcriptions and its targets in vivo

The sequence of the native promoter of GA20ox1 promoter (*Pro-GA20ox1*) and GAMYB (*Pro-GAMY*B) were amplified from genomic DNA, using primers as below: GA20ox1-LUC- F: CTATAGGGCGAATTGGGTACCTCCTATCCCGCTAGATCA CCA, GA20ox1-LUC-R: CGCTCTAGAACTAGTGGATCCCTCTCTCCCCTAGCT ACCTTCT; GAMYB-LUC-F: CTATAGGGCGAATTGGGTACCGCTTGAATTGG GAGGGCAGA, GAMYB-LUC-R: CGCTCTAGAACTAGTGGATCCGCGTCTCA ACTACACCGGA; Construction of the reporter: vector of pGREENII-0080-luc was applied to clone the above amplified promoters through a one-step cloning kit (Vazyme, Nanjing, China). Construction of the effector: CDS region of the GAI transcription factor was amplified and cloned into *pGREENII-62-SK* vector using the followed primers: GAI-SK-F: CGCTCTAGAACTAGTGGATCCATGAAGCGCGAGTACCAAGA, GAI-SK-R: TCAGCGTACCGAATTGGTACCTCACGCCGCGGCGACG by one-step cloning kit (Vazyme, Nanjing, China). Transient expression assay was performed as described previously [51].

### Quantitative real-time PCR

First-Strand cDNA Synthesis was obtained using ThermoScript™ RT-PCR System. UBQ5 was used as an internal standard. Each of the gene was performed with three biological replicates. The primers used for qRT-PCR are listed in Supplementary Table S7.

### Statistical analysis

The data analysis was performed in the Prism 6.0, and the results were expressed as the mean values ± SD (*n* = 3). Student t test at a significance level of *P* < 0.05 was used to analyze the variable data.

## Supplementary Information

**Additional file 1.** 

**Additional file 2.** 

## Data Availability

Sequence data from this article can be found in the GenBank/EMBL data libraries under accession numbers PRJNA737598.

## Abbreviations

DEGs: Differentially expressed genes
G6P: Glucose-6-phosphate
TFs: Transcription factors
GO: Gene ontology
KEGG: Kyoto Encyclopedia of Genes and Genomes
DEU: Differential exon usage
RPKM: Reads Per Kilo bases per Million reads
CIPK: CBL-interacting protein kinase
SUS: Sucrose synthase
SPS: Sucrose phosphate synthase
SPP: Sucrose phosphate phosphatase
