# Supplementary material for: Analysis of gene expression in early seed germination of rice: landscape and genetic regulation
Source: BMC Plant Biol. 2022 Feb 17;22:70. doi: 10.1186/s12870-022-03458-3 (PMC8851807; doi:10.1186/s12870-022-03458-3)
Supplement: Supplementary file 2 — Additional file 2. [file 12870_2022_3458_MOESM2_ESM.docx]

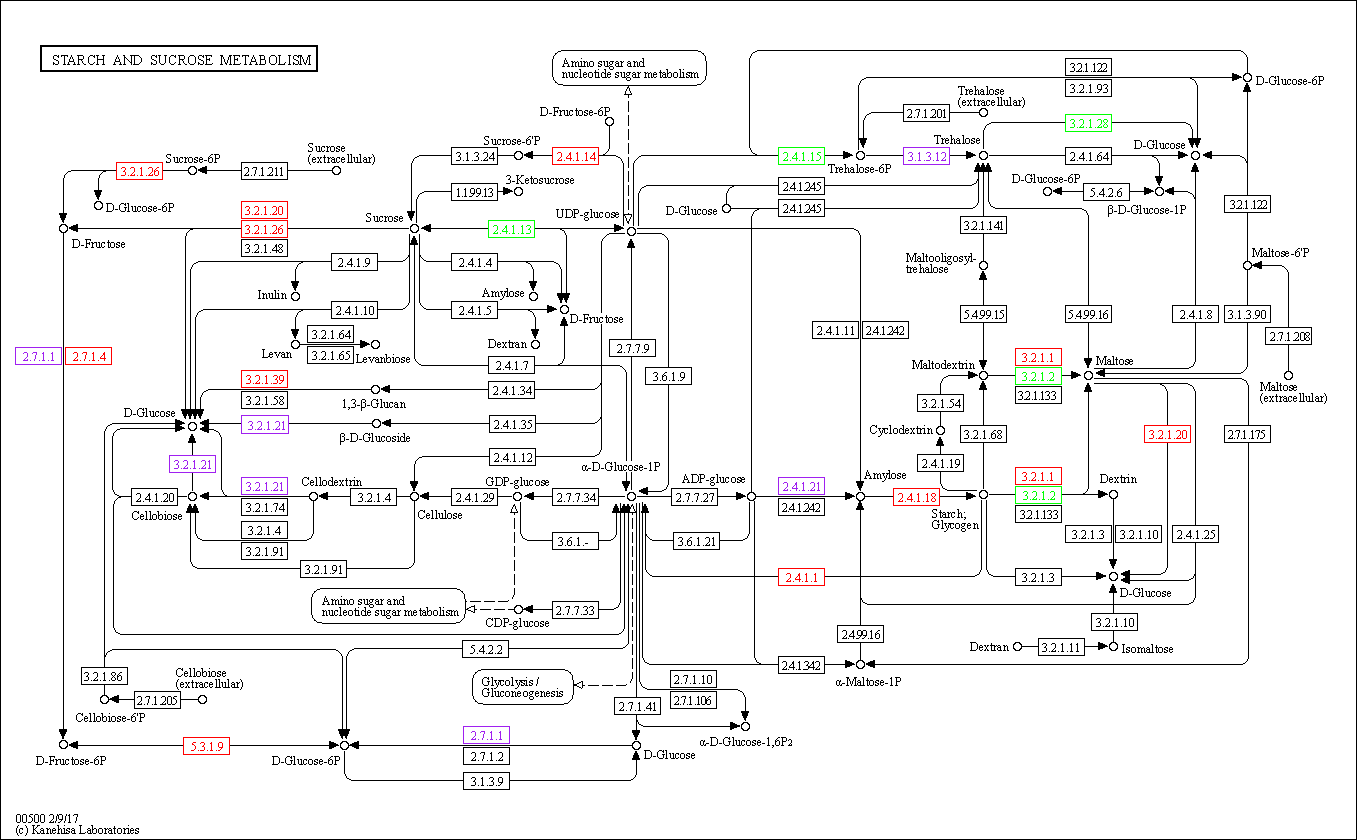


**Supplemental Figure S1 Up regulated genes (red block) and down regulated genes (green block) involved in the pathway of “starch and sucrose metabolism”. (https://www.genome.jp/kegg/pathway.html)**

**Supplemental Figure S2 Differentially expressed genes involved in the hormone signal pathway.**

A, Heatmap of differential expressed genes in the GA singal transduction pathway . B, Differential expressed genes in the salicylic acid (SA) singal transduction pathway. C, Differential expressed genes in the jasmonic acid (JA) singal transduction pathway. D, Transcript levels of ABA responsive transcription factor *ABI5*.

**Supplemental Figure S3 Relative expression levels of *GAMYB*, *bHLH* and *NAC90* validated by qPCR.**


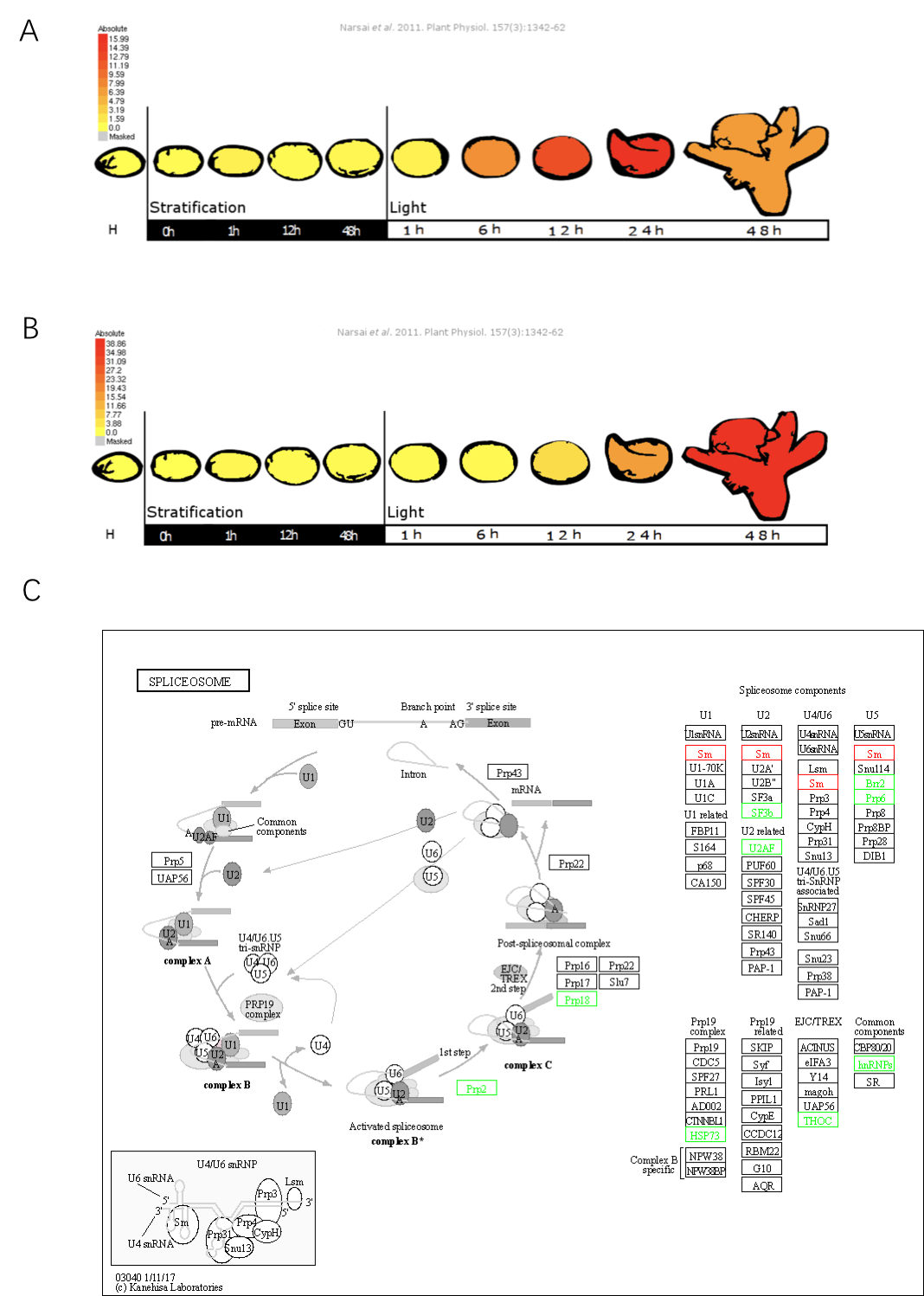


**Supplemental Figure S4 Differential exon usage (DEU) genes and expression pattern of splicing factors.**

A, Gene of OS01g0765000 encoding Cytidine/deoxycytidylate deaminase family protein which showed elevated DEU, displayed similar expression pattern to it’s orthologous genes of Arabidopsis during seed germination. B, Gene of OS02g0653400 encoding transferase family protein which showed elevated DEU, also displayed similar expression pattern to it’s orthologous genes of Arabidopsis during seed germination. C, Expression pattern of splicing factors in the splicesome. Green block is gene down regulated at 24 hours after imbibition. Red block is gene up regulated at 24 hours after imbibition.
